# COVID-19 and mental health in Australia – a scoping review

**DOI:** 10.1186/s12889-022-13527-9

**Published:** 2022-06-15

**Authors:** Yixuan Zhao, Liana S. Leach, Erin Walsh, Philip J. Batterham, Alison L. Calear, Christine Phillips, Anna Olsen, Tinh Doan, Christine LaBond, Cathy Banwell

**Affiliations:** 1 The Research School of Population Health (RSPH), College of Health and Medicine, The Australian National University, Canberra, ACT Australia; 2 The ANU Medical School, College of Health and Medicine, The Australian National University, Canberra, ACT Australia

**Keywords:** COVID-19, Mental health, Australia, Systematic review

## Abstract

**Background:**

The COVID-19 outbreak has spread to almost every country around the world and caused more than 3 million deaths. The pandemic has triggered enormous disruption in people’s daily lives with profound impacts globally. This has also been the case in Australia, despite the country’s comparative low mortality and physical morbidity due to the virus. This scoping review aims to provide a broad summary of the research activity focused on mental health during the first 10 months of the pandemic in Australia.

**Results:**

A search of the Australian literature was conducted between August-November 2020 to capture published scientific papers, online reports and pre-prints, as well as gaps in research activities. The search identified 228 unique records in total. Twelve general population and 30 subpopulation group studies were included in the review.

**Conclusions:**

Few studies were able to confidently report changes in mental health driven by the COVID-19 context (at the population or sub-group level) due to a lack of pre-COVID comparative data and non-representative sampling. Never-the-less, in aggregate, the findings show an increase in poor mental health over the early period of 2020. Results suggest that young people, those with pre-existing mental health conditions, and the financially disadvantaged, experienced greater declines in mental health. The need for rapid research appears to have left some groups under-researched (e.g. Culturally and Linguistically Diverse populations and Indigenous peoples were not studied), and some research methods under-employed (e.g. there was a lack of qualitative and mixed-methods studies). There is a need for further reviews as the follow-up results of longitudinal studies emerge and understandings of the impact of the pandemic are refined.

**Supplementary Information:**

The online version contains supplementary material available at 10.1186/s12889-022-13527-9.

## Background

The outbreak of COVID-19, an infectious disease causing severe acute respiratory syndrome, led the Director-General of the World Health Organisation (WHO) to declare a public health emergency of international concern on the 30^th^ January 2020 [72]. By April 2021, the disease had spread to almost every country around the world, and caused more than 3 million deaths [74]. The pandemic has triggered enormous disruption in people’s daily lives and has undoubtedly had a widespread and profound global impact.

Australia has managed to date to achieve low total numbers of local infection, partly because of its geographic isolation (i.e. all borders are surrounded by sea) and also because of early interventions to contain the virus. Following the first confirmed case on the 25^th^ January 2020 [34], the Federal Government quickly introduced border controls, quarantine measures and urged the public to take precautions in response to the virus. By March 2020, a series of stringent containment measures were put in place by the state and territory governments to stop the spread of the virus and protect people’s lives. These included requirements to stay at home (except for specific reasons), business closures, restrictions on social gatherings and interstate travel, as well as a ban on all international travel. Residents in the state of Victoria experienced particularly stringent restrictions (e.g. a nightly curfew, a 5 km-limit for all activities, and mandatory mask-wearing [6]) during a second wave of COVID from June-October, 2020. To date, these restrictions have proven to be successful at reducing the transmission of the virus in Australia [16, 13]. However, they have come at a considerable economic and health cost to individuals, businesses, communities and the nation. Government data shows that during June-July, 2020, the Australian Gross Domestic Product fell by a record 7% and the unemployment rate hit 7.5%—the highest it had been in over 20 years. Reassuringly, after July, the Australian economy started to improve in all states except Victoria [3].

Despite the successful management of the pandemic to date and the ongoing economic recovery, there are indications that Australians’ mental health declined in the early months of the pandemic and that this reduction has been somewhat sustained. Data from the Australian Bureau of Statistics (ABS) shows that in January 2021 22% of Australians reported that their mental health was ‘worse’ or ‘much worse’ than in March 2020; comparatively only 0.1% of people in Australia have been infected with COVID-19. Similarly, 21% reported that their mental health was ‘fair’ or ‘poor’ in January 2021—higher than the 14.4% who reported this in July 2020 [2, 4]. Although this self-report data is not based on validated mental health measures, it demonstrates the importance of investigating the widespread and potentially enduring impact of the pandemic on mental health in Australia. Mental health experts have stated that increases in mental health problems are likely due to risk factors attributable to the virus itself (e.g. fear of contracting the virus, concerns about the lack of treatment options and/or being in a high-risk group for mortality, and uncertainty about when the virus will be controlled) as well as risk factors attributable to the lockdowns aimed at combating the virus (e.g. interrupted daily routines, unemployment and underemployment, loss of income, reduced social support, financial distress, and loneliness) [38]. The latter are well-established risk factors for poor mental health generally, let alone within the complex context of a global pandemic [50, 32].

The Australian context is unusual in terms of the focus on individuals’ and communities’ mental health in 2020. In part, because the prevalence of COVID-19 has been relatively low in Australia compared to other countries, discussion regarding the more distal mental health impacts of COVID has been prominent alongside concerns about the proximal physical impacts. Justifiably, the research community (and the media) in Australia has paid tremendous attention to the potential mental health impacts of the outbreak. An influx of studies have been conducted in the past year (mainly from March to September 2020) to understand people’s experiences and gauge any increase in mental health problems during the pandemic. While many of these studies are still ongoing, numerous results have been published reporting on the prevalence and severity of mental health problems during this time (mostly common experiences such as psychological distress, depression and anxiety), and the vulnerability of different groups. For context, it is also important to note that the COVID pandemic closely followed the Black Summer bushfires. From September 2019 to February 2020, large swathes of Australia were burnt, accompanied by destruction of life, property, the natural environment and wildlife [11] (although most COVID-focused studies have not considered the population’s possible lingering emotional responses to the bushfires).

Despite the influx of research activity in Australia investigating mental health during 2020, comprehensive summaries of what has been done and what has been found are scarce (for an international review and meta-analyses see Prati & Mancini [56]). Given it has been over a year since the outbreak began, the current scoping review provides a timely summary of the Australian research conducted in 2020 during the early phase of the COVID-19 outbreak. The review also aimed to identify gaps in research activities, knowledge and understanding of how the pandemic is affecting Australian’s mental health.

## Methods

### Study design

In this review, the use of the term ‘mental health’ goes beyond the presence/absence of diagnosed mental illness and instead focuses on the most common psychological symptoms experienced in the community, such as distress, anxiety, and depression. Because this review aimed to be inclusive, and also considering much research regarding the pandemic is ongoing (with some research reports and online pre-prints not yet available in peer-reviewed scientific journals), we deemed a descriptive broader scoping review more appropriate than a traditional systematic review [44, 64]. This review follows the PRISMA-ScR checklist, an extension of the PRISMA statement for conducting scoping reviews [66, 51].

### Eligibility criteria

While this scoping review was necessarily broad, clear well-defined eligibility criteria and research questions were still required. Following the JBI recommendations [51] we define our *population* as Australians, our *context* as Australia during the first 10 months of the COVID-19 pandemic, and our *concept* as mental health prevalence (or outcomes) and risk factors during this window of time.

Publications (reports, non-reviewed pre-prints of papers and peer-reviewed articles) were eligible to be included if they were focused on mental health during the COVID-19 pandemic, reported original research findings/results (i.e. media releases, editorials, opinion pieces, commentaries, protocol papers or general text summaries within reports (with no detailed findings) were excluded), were conducted within the Australian population, and were written in English.

### Literature search and data extraction

Searches of the literature were conducted between August-November 2020 to capture research with a focus on COVID-19 and mental health in Australia. The search included three elements:

1. Four databases (PsycINFO, PubMed, Scopus and Web of Science) were searched using key words to capture published peer-reviewed articles focused on COVID-19 and mental health in Australia. These keywords were COVID AND ("mental health" OR "psychological dis*" OR "mental dis*" OR depress* OR anxiety OR wellbeing OR well-being OR "well being" OR worr* OR fear OR lonel* OR "alcohol use" OR "substance use” OR stress OR confus* OR anger OR optimism OR pessimism OR "mental ill*" OR mood OR panic) AND Australia*. The search was generally within the title and abstract field (in some databases, keywords and author information were also included). The document type was limited to “article” where possible so that other types of publications such as reviews, study protocols, editorials, commentaries, viewpoints, letters to editors, and dissertations, were excluded.
2. The online search engine Google was searched using the phrase “COVID mental health research survey Australia” to capture research findings not yet published in scientific journals. The results were limited to records within one year, verbatim, and pages published in or originating from Australia. Reports, online papers and pre-prints that included mental health/wellbeing measures or interview questions (and sufficient information about study methods) were identified and recorded. In addition, we checked the reference lists of identified publications and reached out to our existing research networks to identify relevant pre-prints or recently accepted publications.
3. All the records in the databases for the Research Tracker and Facilitator for Assessment of COVID-19 Experiences and Mental Health project [14] were checked for any additional studies not already identified. This project aims to track research being undertaken on COVID-19 and mental health by Australian researchers.

## Results

### General description of studies included

**Fig. 1:**
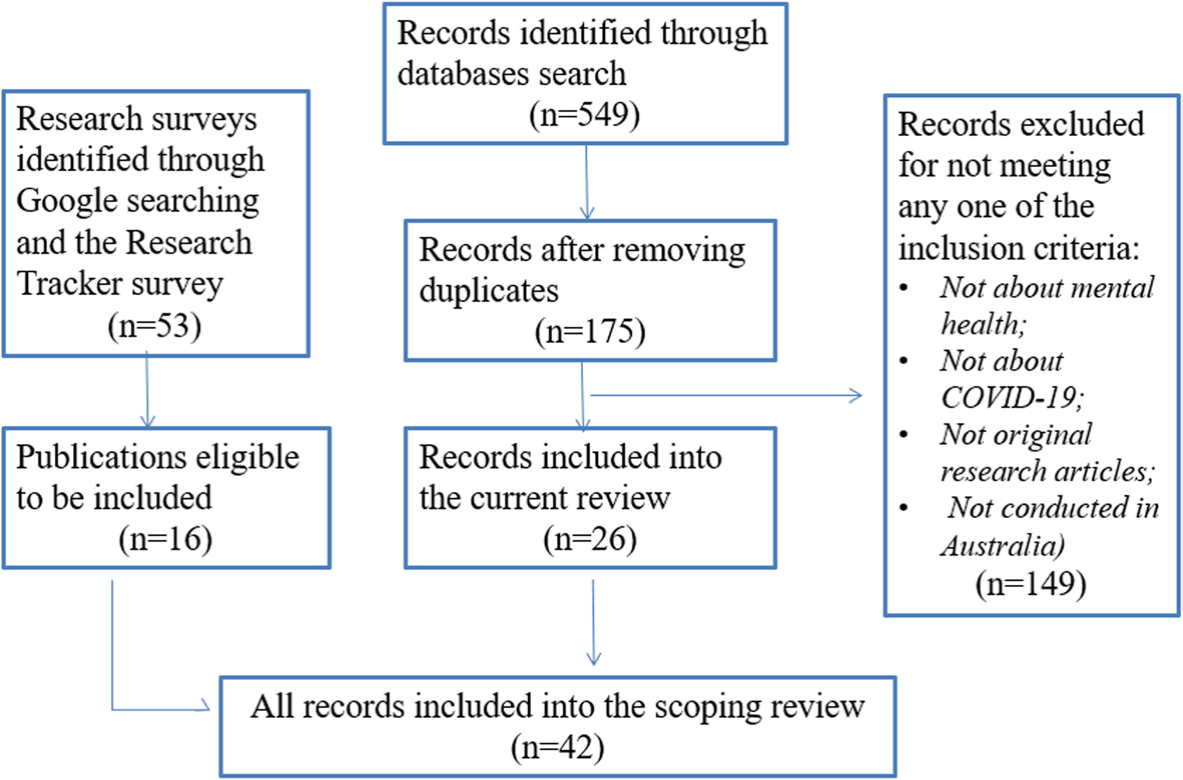
Search and selection process for the review

The search and selection process is outlined in Fig. 1. As the manual search of reference lists did not yield any more records beyond the records identified through other search methods, this was not specified in Fig. 1. The records identified through the database searches were reviewed by two researchers (YZ and LL) independently. Any disagreements regarding the eligibility of articles were resolved via broader discussion with the project team. Overall, 42 articles were identified as eligible for inclusion in the scoping review. Two reviewers (YZ and EW) independently assessed the full-texts of the 42 articles and extracted and recorded relevant data (including sample characteristics, whether the study included pre-COVID comparisons, mental health outcomes and measures, study key findings, and any main risk or protective factors identified). All discrepancies regarding data extraction were resolved through discussion.

The characteristics of the 42 included studies are outlined in Tables 1 and 2 (see Additional file 1).

#### Study time-frame and geographical coverage

The majority of the eligible studies were conducted between the end of March and early June 2020, covering the time period when the whole country was under stringent stay-at-home measures, with strict restrictions placed on social gatherings. Seven studies included data collected after this period, when the restrictions were beginning to relax across Australia (except for Victoria) [9, 10, 30, 36, 39, 40, 57]. All but one [39] of these seven studies included data from every state including Victoria after the second wave’s containment measures. However, Griffiths et al. [30] was the only study that made direct comparisons between Victoria and the rest of Australia.

#### Study populations

Out of the 42 research studies, 12 were conducted among the general Australian adult population, while the remaining 30 focused on a specific group within the population (e.g. parents of young children, health workers, people with an existing health or mental health condition, or young people). The characteristics and key findings for the general population studies are summarized in Table 1 and for specific group studies in Table 2. Three studies [10, 52, 68] drew a subsample of data from surveys conducted among the general population. However, because the aims and findings of these studies focused on specific subpopulation groups, they were included as research conducted among specific groups.

#### Pre-COVID comparisons

Of the total 42 studies, nine studies were longitudinal or repeated cross-sectional and had data collection points covering the time period before and during the COVID-19 outbreak (with comparative data collection methods and mental health measures employed) [7, 8, 15, 22, 39, 43, 63, 65, 67]. These studies were more robustly able to compare participants’ mental health during the COVID-19 pandemic to a pre-COVD level. In other words, the evidence provided in these studies was higher quality than other studies with no baseline pre-COVID comparison. Ten further studies compared the results of their studies to norms or results of similar studies conducted before the pandemic. Four studies asked the participants to self-report on whether, and to what extent, their mental health had changed since the onset of the pandemic (these studies are susceptible to recall misjudgements). Several studies used more than one mental health measure and the pre-COVID comparison for each measure sometimes varied. Twenty studies did not report any pre-COVID comparison data, making it difficult to draw confident conclusions about changes in mental health due to COVID.

### Research on the general population in Australia

#### Study sampling and data sources

In the 12 general population studies (Table 1), the participants were usually required to be aged over 18 and currently living in Australia. Four of the 12 studies were based on representative samples of Australian population – 1 & 2. ANUpoll study (Life in Australia™)^1^ [7, 8]; 3. Taking the Pulse of the Nation Survey^2^ [9]; 4. The Australian National COVID-19 Mental Health, Behaviour and Risk Communication (COVID-MHBRC) Survey [18]. Six studies recruited participants online via social media (e.g. through Facebook advertisements) – 1 & 2. Fisher et al. [27] and Owen et al. [48] drew data from the Living with COVID-19 restrictions in Australia survey^3^; 3. Rossell et al. [58] used data from the COVID-19 and you: Mental health in Australia now survey (COLLATE)^4^; 4. Gurvich et al. [31] used data drawn from the COVID-19 and Mental Health Survey^5^; 5. Newby et al. [45] used data from the Mental Health and Coronavirus Study conducted by UNSW and the Black Dog Institute (approval number 3330); 6. Survey data used by Stanton, To & Khalesi et al. [62] (approval number 22332). The sample representativeness when recruiting participants via online platforms varies greatly in published research [53]. It is generally accepted that studies based on random and/or representative samples are higher quality with more generalisable findings. However, online methodologies are considered feasible and efficient for broadly summarising population experiences and for correlational research, as they provide timely access to a significant number of individuals [40]. The two remaining studies in Table 1 [21, 25] were based on analyses of online content. Given the ubiquity of internet use, analysing online content offers researchers an avenue to understand public sentiments and opinions [21, 25].

#### During-COVID/Pre-COVID study comparisons

Most of the surveys investigating the COVID-19 outbreak and mental health have collected, or intend to collect, follow-up data to understand changes in the public’s experiences and mental health symptomology as the pandemic evolves, but currently available publications mostly report baseline data. In other words, the majority of studies are cross-sectional and the longitudinal results are not yet available. Out of the 12 studies included in Table 1, four report changes in participants’ mental health over time during the pandemic. These studies correlate changes in mental health symptomology with varying case rates of COVID-19, as well as changes in social and economic policies and other life circumstances in the first few months of the pandemic [7, 9, 21, 25].

In terms of pre-COVID comparisons, we identified no studies tracking mental health from pre-COVID and into the COVID period using the same sample/cohort over time. However, six of the 12 studies made comparisons between current COVID results and results from a pre-COVID sample in Australia. Biddle et al. [7] and [8] compared their current results with previous waves of the same survey, although the same cohort of respondents was not tracked individually. Four studies compared their results with findings from various representative studies conducted prior to COVID [9, 18, 27, 58]. These comparisons provided some information about whether, and how, people’s mental health changed during COVID, but the comparisons are less rigorous than if pre-COVID data were available from longitudinal cohort studies tracking temporal changes in individuals.

#### Mental health outcome measures

Studies generally focused on psychological distress, depression and anxiety. These mental health problems were primarily examined using validated psychometric scales – demonstrating good quality, robust measurement. The most common measures included the Kessler 6 (K6) scale (used by Biddle et al., [7, 8] as an indicator for general psychological distress; Patient Health Questionnaire-9 (PHQ-9) (used by Dawel et al. [18]; Fisher et al. [27]; Owen et al. [48]) to assess depression symptoms, suicidality and eating patterns; Generalized Anxiety Disorder-7 (GAD-7) (used by Dawel et al. [18]; Fisher et al. [27] to measure anxiety and irritability; and the 21-item Depression Anxiety Stress Scales (DASS-21) (used by Gurvich et al. [31]; Newby et al. [45]; Rossell et al. [58]; Stanton et al. 62]) to measure dimensions of depression and anxiety symptoms. Gurvich et al. [31] also reported on suicidal thoughts using the relevant items in Beck Depression Inventory (BDI). Among the two studies analysing online content, Du et al. [21] selected the terms “fear”, “panic”, “worry” to represent fear-related emotions as they showed high consistency with each other, while Ewing & Vu [25] harvested public sentiments through researchers’ interpretations of the tweet data from Twitter.

#### Overall study findings

The results of the four nationally representative studies (Biddle, et al. [7, 8], Botha et al. [9], Dawel et al. [18] all showed an increase in mental health problems compared to pre-pandemic published statistics. Three of the remaining general population studies also found an elevation in mental health problems when comparing their results with pre-pandemic norms [27, 45, 58]. Du et al. [21] tracked the internet searches for fear-related emotions, protective behaviours, health-related knowledge, and panic buying by Australian throughout March, and Ewing &Vu [25] analysed 3-weeks of tweets by Australian in April. They both found a decline in positive emotions, which matched the deterioration of the COVID-19 situation over time. The three studies by Gurvich et al. [31], Owen et al. [48] and Stanton et al. [62] had no pre-COVID comparisons, and provided no evidence about whether mental health deteriorated during the pandemic. Instead, these studies identified a series of risk and protective factors for mental health during COVID-19. Despite the reports of pessimism in the population, some optimistic feelings were also identified – Biddle et al. [ 8] found a significant increase in social cohesion and trust to fellow Australians in the population and Fisher et al. [27] found that on average Australians were optimistic about the future.

Several studies identified demographic and socio-economic characteristics associated with mental health during COVID-19. For example, Newby et al. [45], Biddle et al. [7] and Dawel et al. [18] all found that younger people reported poorer mental health during the pandemic relative to older groups. Those who experienced job loss, reductions in work hours, and financial hardship during COVID were also more likely to record mental health problems (e.g. [7, 9]). Another important factor was pre-existing mental health conditions. Participants with a prior mental health diagnosis were more likely to report worse mental health during COVID-19 [18, 45, 58, 62].

Studies also showed that people who were worried about contracting COVID-19 were more likely to report poorer mental health [27, 45, 48]. Surprisingly, Dawel et al. [18] found that direct COVID-19 exposure was not associated with mental health problems. Instead, impairments in work and social functioning and financial distress due to COVID-19 were more strongly associated with poorer mental health. Dawel et al.’s study [18] also considered the experience of bushfire exposure during the 2019–2020 fires. The results showed that exposure to the fire was not associated with mental health symptomology, but exposure to the bushfire smoke was associated with decreased wellbeing.

### Research on specific subpopulation groups

The 30 studies with a focus on specific subpopulations included 25 quantitative studies (with the majority based on survey data and five based on administrative data), four qualitative studies and one mix-method study. Two of the four qualitative studies (Digby et al., 2021; [19, 24]) reported the qualitative findings of mixed-methods research, with the quantitative findings reported elsewhere.

#### Study samples and populations of interest

Of these 30 studies, 20 studies collected data from participants across the nation (although one comprised largely of people living in Victoria (88.2%)) [57]. Only Sollis et al. [61] and Broadway et al. [10] were based on survey data analysed from nationally representative samples, and Johnston et al. [ 36] pre-stratified their data/sample to approximate a nationally representative sample. The remaining ten studies focused on specific states or cities. One focused on South Australian [67]; one on Queensland [39]; two studies were conducted in Western Australia [22, 41]; and two studies in Sydney or New South Wales [43, 60]. Four studies were conducted in Melbourne or Victoria [15]; Digby et al. 2020; [20, 33].

People with a particular vulnerability were a major focus of these studies. They included patients presenting to and/or staying in hospital due to poor health or mental health in the study period [15, 22, 60]; people with a pre-existing physical or mental health disorder [52, 68]; and people accessing mental health services [63, 65, 67]. Leske et al. [39] studied suicide rates and motives during the pandemic. Hospital staff, whose physical and mental health may have been more vulnerable during the pandemic, were the population of interest in three studies (Digby et al., 2021, [19, 20, 33]. Other potential participant vulnerabilities included being an adolescent or young adult [40, 41, 43], in self-isolation/quarantine [35], living alone [46] and having higher dysmorphic concern [55].

Families with young children were considered vulnerable and therefore a population of interest in nine studies. Six studies drew data from the COVID-19 Pandemic Adjustment Survey which was conducted among parents of children under the age of 18 (see Table 2). Two studies drew data from other nationwide surveys [36, 10]. Additionally, Chivers et al. [17] conducted a qualitative research on new and expecting parents.

#### Pre-COVID/ during-COVID study comparisons

As indicated in Table 2, 15 of the 30 studies reported on changes in mental health and other wellbeing indicators before and during the COVID-19 outbreak. Most studies investigating specific populations were cross-sectional and compared current results with the results or statistics from pre-COVID studies that used similar samples (or comparable admissions/administrative data). Other studies asked participants to self-report on the differences in their mental health before and during the pandemic. Four studies reporting administrative data from health services [15, 22, 63, 65] selected data collected during the corresponding period of 2019 as their pre-COVID comparisons (to avoid the period immediately before the pandemic when Australia experienced the severe bushfire crisis). One longitudinal study tracking the same cohort of participants [43] adopted a cut-off date to compare mental health before and after the implementation of the COVID-19 restrictions. Separate from the pre-COVID comparisons, four studies [15, 22, 30, 63] compared mental health across multiple time points during the pandemic, linking changes in participants’ mental health to changes in case rates for COVID-19 in Australia.

#### Mental health measures

Similar to studies focused on the whole general population, most of the subpopulation studies measured participants’ mental health and wellbeing using validated scales such as the K6, K10, PHQ-9, GAD-7 and the DASS-21. A series of other mental health measures were also adopted (see Table 2). Apart from the validated mental health measures, behaviours related to mental health, including eating and exercise behaviours [52], and appearance-focused behaviours [55], were also adopted as mental health indicators. Several studies examined public or administrative records, including emergency department presentations [15, 22], suicide registers [39] and website visits and call centre traffic for mental health services [65, 63]. A small number of studies did not use validated measures and instead asked participants to self-report on their mental health, lowering the quality of mental health measurement in these studies (e.g. [10, 35, 36, 41, 43]). None of the sub-group studies assessed the widespread and likely traumatic impact of the 2019–20 bushfires (a significant individual and community-level pre-pandemic vulnerability).

Five studies qualitatively assessed participants’ descriptions of their experiences and feelings during the COVID-19 pandemic [17], Digby et al., 2021; [19, 24, 46, 60] to gain a deeper understanding into participants’ psychological wellbeing in relation to their specific contexts. Of the five studies, Chivers et al. [17] analysed posts related to COVID-19 in an online parenting forum to understand perinatal distress. Shaban et al. [60] conducted bedside interviews of COVID-19 patients to explore their lived experiences and perceptions. The other three studies added open-ended questions asking about participants’ concerns related to COVID-19 in their surveys.

#### Overall study findings

In general, the studies investigating specific subpopulation groups showed similar patterns to the findings of the studies on the general population – mental health and wellbeing deteriorated with the emergence of the COVID-19 pandemic and associated restrictions. This trend is consistent across the different populations of interest. However, it is also apparent that important population groups, such as Indigenous and CALD (Culturally and Linguistically Diverse) groups were not researched, limiting our knowledge for these groups. Psychological distress was reported widely among hospital staff in the two studies that measured hospital workers’ mental health [20, 33]. Three studies focusing on adolescents and university students consistently showed higher psychological distress and lower subjective wellbeing since the COVID-19 outbreak [40, 41, 43]. Studies focusing on parents with young children identified a range of mental health challenges and risks during the COVID-19 period, and the three studies that included a pre-COVID comparison indicated that psychological distress increased [10, 70, 71]. The themes identified from the qualitative studies differed as they were specific to the experiences of each subpopulation group. However, participants in these studies acknowledged the impact and the challenges brought by the COVID-19 pandemic and expressed worry and concerns (refer to Table 2 for details).

The two studies [30, 63] reporting on participants’ mental health several times across the pandemic showed similar results to Biddle et al.’s [7] study of the general population. Griffiths et al. [30] focused on working adults and Staples et al. [63] focused on consecutive users of digital mental health services during the pandemic. Corresponding with Biddle et al. [7], both studies found that declines in mental health appeared to be more significant during March to April, and then improved in later months (returning normal levels) (except for the Victorian participants in Griffiths et al. [30]).

In contrast to the consistent findings from survey data showing increases in common mental health problems (i.e. psychological distress, depression and anxiety), two studies analysed data on emergency department (ED) presentations during the pandemic and showed varying results. Cheek et al. [15] found that mental health presentations potentially increased,while Dragovic et al. [22] found that the total number of mental health presentations decreased and that the trend varied depending on the reasons for the presentation. A decrease in ED presentations is not surprising given that face-to-face access to many health services declined during the pandemic (as people restricted their mobility) [5] – and thus, actual service use during this time does not likely reflect the need for services in the community. Importantly, according to data from AIHW [5], mental health related services, particularly services delivered online or via phone showed heightened service usage since the restrictions were introduced. The contrast between the two studies is likely because they were based on data from two different states with different COVID-19 responses, and Cheek et al. [15] only included paediatric patients.

In terms of suicidal intention, plans or behaviours, data from Queensland showed no change in suspected suicides [39] and in Western Australia, the presentations to emergency departments due to suicide or self-harm decreased significantly during this period [22]. On a national level, those who accessed digital mental health services during the pandemic also showed no changes regarding suicidal thoughts or plans [63].

Several potentially positive experiences related to the COVID-19 situation were identified from existing studies. Many individuals and families practicing isolation/social distancing reported some “silver linings”, such as strengthening relationships with their families, enjoying spending time at home, and developing new hobbies [24, 35]. Patients with COVID-19 who were in isolation also reported some positive factors [60]. For example, although patients reported that they were disconnected from the outside world, lost track of time, and had limited mobility, some saw this as a reflection of the professionalism and quality of care provided. This enhanced their confidence and helped to ameliorate their initial concerns about being infected. Positive experiences were also identified as potential indicators of resilience and helped to mitigate the negative effect of the pandemic and restrictions on mental health [20, 35, 42, 24]. For example, Oliva & Johnston’s study [ 24], showed the mental health benefits of having a dog during the lockdown, likely because it encouraged exercise and provided an opportunity to socialize with other people.

Several studies made comparisons between specific population groups and the general population, or other population groups. These studies provide insights into which population groups might be at greater risk of experiencing mental health problems, and what factors were protective during the pandemic. Specifically, Broadway et al. [10] showed the protective effect of having two earners in the family in times of uncertainty. Phillipou et al. [52] found that individuals previously diagnosed with eating disorders experienced more mental health problems compared to the general population while people with high and low dysmorphic concern displayed different psychological and behaviour responses to the shutdown of the beauty industry in the COVID-19 lockdown [55].

## Discussion

In summary, we found that Australians in general experienced poorer mental health during the early stages of the pandemic in 2020 compared to pre-COVID. However, the absence of robust longitudinal cohort studies with pre-pandemic baseline data with makes this difficult to conclude definitively. Despite variation in the prevalence of and responses to COVID in individual countries, internationally research similarly indicates there has been a consistent deterioration in mental health and wellbeing levels around the world (see Findlay et al. [26] (Canada), Fitzpatrick et al. [28] (US), Pierce, Hope & Ford et al. [54] (UK). For example, the results of a meta-analysis [56] of longitudinal studies and natural experiments regarding the psychological impact of COVID-19 pandemic lockdowns internationally, aligns with our findings, showing an increase in psychological symptoms such as depression and anxiety, but no changes in suicidal risk. However, it is worth mentioning that all studies above were conducted in relatively high-income countries. Low-to-middle income countries have experienced even greater impacts during the pandemic, because of their inadequate and underprepared health systems and the uncertainty of their economies. Therefore the mental health impacts of COVID-19 are possibly more serious in the low-to-middle income countries and worthy of specific attention [1, 12].

Apart from this general trend, some other key issues regarding the impact of the COVID-19 pandemic on mental health were also evident from the research findings. First, a series of demographic and socio-economic characteristics were identified as risk factors for adverse mental health outcomes. Most clearly, mental health and wellbeing levels seemed to deteriorate in younger age groups – while adolescents and young adults are at greater risk of poor mental health at any time (i.e. outside of pandemic conditions) the deterioration in their mental health during COVID appeared greater than for older age groups [7]. One explanation is that age is associated with other mental health risk factors that were heightened during the pandemic – such as employment and financial status. In April 2020, the underemployment rate in Australia was 13.8% while the youth underemployment rate hit 23.6% [3]. Along with employment and financial insecurity, young people are also more likely to have precarious housing and be more reliant on social and peer support which diminished during the pandemic [69]. As a consequence, it appears there has been a disproportional impact on younger adult’s mental health, despite their relative physical robustness [73]. Another important risk factor identified was pre-existing mental health problems. Earlier in 2020, Galletly [29] stated that the pandemic would be a difficult time for people with chronic mental illness. This is echoed by research showing that participants with a prior mental health diagnosis had poorer mental health during the pandemic – however the lack of studies reporting pre-COVID comparative data makes it difficult to determine the extent to which mental health decline for this group comparative to those with no pre-existing mental health problems.

The current review found that people reported some positive mental health and wellbeing experiences that emerged during the early stages of the pandemic. Potentially positive experiences reported by the participants in the reviewed studies included strengthening relationship with family and increased confidence in healthcare system [24, 60]. Identifying the positive aspects of peoples’ experience during this challenging time is as important as identifying risk factors in terms of grasping a holistic understanding of what approaches and strategies are most useful to mitigate the negative impact of the pandemic on mental health.

### Shortcomings in the research response

The current scoping review demonstrates that many Australian mental health researchers, like researchers internationally, responded rapidly to the pandemic. While this swift response captures a highly valuable snapshot of the impacts of this worldwide disaster, there are shortcomings in terms of design and the reliability and validity of findings. One key gap highlighted in this review is the lack of longitudinal studies with comparative pre-COVID data from the same cohort. Consequently, conclusions about how mental health changed over the course of the pandemic (from pre-pandemic levels), how people adapted during COVID, and whether trajectories varied for different groups are currently limited. A number of important national Australian studies (longitudinal and repeated cross-sectional) are yet to release data collected towards the end of 2020 (e.g. the Longitudinal Study of Australian Children wave 9C1; the ABS Intergenerational Health and Mental Health Study) – we expect these and other studies still to be published will go some way to addressing this knowledge gap. A further shortcoming is that the impact of the 2019–2020 Australian bushfires has rarely been considered.

The small number of qualitative and mixed method studies indicates another gap in the available research. There is value in adding qualitative research components to the mix that can elucidate contextual factors and lived experience particularly for specific and vulnerable groups which may assist in better provision of services to them. As COVID-19 is a novel virus leading to unprecedented challenges and experiences, qualitative research may contribute to a deeper understanding of the complexities (and emerging issues) of mental health and wellbeing pathways during the pandemic, and its potentially lasting impact on mental health once the pandemic has subsided.

These possibilities suggest that we need to fund good quality longitudinal research, as well as turn to rigorous and multi-faceted research. There is a need to gather baseline and follow-up data (including the use of administrative data, longitudinal, mixed-methods studies, and in-depth qualitative research). On a practical note, while the practicalities and mechanics of research are not the focus of the current review, it is important to note that the pandemic has revealed some of the barriers to conducting high quality mental health research that is responsive and has longevity. The time sensitivity of the pandemic, and its rapidly evolving nature highlighted delays related to need to for prompt ethics clearances across multiple institutions in Australia (under-resourced ethics committees were inundated with requests that needed to be expedited). The formal requirements of funding bodies are not well suited to rapidly evolving pandemics either, with funding for COVID-19 mental health research announced in November 2020 after the most restrictive lockdowns had ended. While Australia is a success story compared to similar wealthy western nations, the mental health impacts of COVID-19 (and the current gaps in this body of research) suggests that efforts to address current research practices and resource constraints may improve the country’s responsiveness to comprehensively study future challenges.

### Research still to come….

The studies included in this review were conducted generally between April–May 2020. However, the COVID experience in Australia and worldwide is rapidly evolving: it has been contained in some Australian locations while additional outbreaks have occurred elsewhere. It is anticipated that Australian research results from studies conducted in the second half of 2020 and early 2021 will be different from those reported in the current review as efforts to contain the virus have been also evolving across the states and territories. The vaccine program rollout, currently being implemented, may have a significant impact. Research on the long-term mental health effects of disasters suggest that people’s responses evolve considerably [59]. However, the health nature of this pandemic may differentiate it from natural disasters, and comparative literature is not currently available.

While most existing studies show that COVID-19 containment measures have impacted negatively on the mental health of the general population and on specific vulnerable groups, it is anticipated that the population’s mental health outlook will improve as the vaccination program takes hold and lockdown measures are no longer needed [30]. However, the discontinuation of the national Job-keeper program (a federally funded program paid to businesses to keep their employees) and the Coronavirus Supplement payment for Job-seekers (an unemployment payment) [23] by end of March 2021 may trigger job and income losses, leading to declines in mental health for some. Financial insecurity is an important risk factor for poorer mental health—the Taking the Pulse of the Nation survey showed mental distress (depression or anxiety) was closely aligned with financial stress throughout 2021 [10]. Concerns remain for those with pre-existing mental health conditions, for those who may experience financial hardship over a long period, and for those who experience future lockdowns. For example, it appears that the mental health of residents in Victoria varied from the rest of Australia [30] as they were subjected to a second lengthy and severe lockdown period when the virus re-emerged that delayed re-entry to employment, schooling and services.

The COVID-19 pandemic may have a delayed impact on mental health in subpopulation groups in myriad interactive and cumulative ways. One example is the mental health of those who were pregnant during the early phases of the pandemic, who in 2021 will have infants and be in the postnatal phase and may have added vulnerability to postnatal depression and anxiety. In addition, as we note above, some vulnerable population groups are under-represented in the existing studies with implications for the management of the pandemic. For example, media reports at the time suggested that some CALD and socially and economically disadvantaged groups may have had different COVID-19 experiences and may have missed out on mainstream messaging; consequently, there may be discrimination that impacts the mental health for different ethnic groups for some time to come. The mental health status of healthcare workers, who have been on the frontline of this crisis, also requires further attention from the research community. The existing studies on the mental health of healthcare workers identified in this review were only conducted among hospital staff in several health services in Melbourne – not nearly enough to cover the experience of this population group in Australia. Fear of transmitting the virus to family, community perception of frontline workers as potential disease carriers, extreme workloads, limited availability of protective equipment and moral dilemmas have all added extra burdens to the mental health of the healthcare workers (Digby et al., 2021) [19]. A systematic review and meta-analysis of studies conducted in other countries has found high prevalence of mood and sleep disturbances among this specific group [49]. These future possibilities and identified research gaps demonstrate the need for ongoing research to better understand what happened to mental health both during the pandemic phase and in the aftermath.

### Limitations

As noted, there are a number of limitations to this scoping review that need to be briefly acknowledged. The first relates to the rapidly changing and emergence of new published results. This review only provides a snapshot of the research available during the period when the existing literature was searched and it is possible that some information published online has been missed. Further updated reviews need to be conducted to continue to synthesize research findings. Second, while the current review did not perform a quality rating of the studies included in the review, discussion of study quality is included throughout and Tables 1 and 2 list detailed information about the characteristics of each study—including document type, sample size and representativeness, as well as whether pre-COVID comparisons were made. This information provides a reference for making judgements about the strengths and weaknesses (quality) of each study. We do conclude that studies published in peer-reviewed journals, based on a nationally representative sample of Australian population, with a pre-COVID comparison sample from the Australian population are the highest quality. We also make the point that prospective longitudinal studies including baseline (pre-COVID) data from the same sample or cohort are the most robust, but are rare. Third, an analysis of publication bias was not undertaken given that the body of literature is still so new – an analysis of publication bias that extends to considering those vulnerable groups that may have been missed (or difficult to access during COVID-19) would be worthwhile once a more substantial body of literature exists.

The review does not provide detailed data on prevalence rates and statistical associations for each study as many of them did not provide this information. Therefore, we instead aimed to scope the breadth of research conducted and provide a narrative overview (in the text and the Tables) of the findings. Future reviews will provide a comparative summary of the prevalence rates and associations (such as meta-analyses), once this information is obtained. Although the range of differences between studies (e.g. measures used, timing of survey within 2020) that we have observed is likely to make it challenging to combine the data to obtain comparative estimates.

## Conclusion

The current scoping review provides a detailed record of the studies published online and in the academic literature investigating mental health during the COVID-19 pandemic in Australia. Our findings suggest that despite the comparatively low prevalence of the disease in the population, mental health problems (i.e. psychological distress, anxiety, depression, poor wellbeing) increased during the early part of the COVID pandemic in 2020. This finding points to the need to focus on mental health problems once the physical health impacts are reduced in countries where the pandemic has been widespread. However, limitations associated with many of the studies in the review, preclude reaching a more definite finding. Young people, those with fewer socio-economic resources and those with pre-existing mental health conditions showed the strongest associations with poor mental health during this time. The review highlights the importance of considering particular vulnerable groups, including health and hospital workers, those in quarantine or isolation, adolescents, parents of children, and people with a pre-existing mental health condition or who were accessing services. Heightened impact on these vulnerable groups suggests that policy attention needs to be given to their economic and psycho-social health to reduce the pandemic’s potentially long-lasting regressive effect. There is a need for further reviews as the follow-up results of longitudinal studies emerge and estimates and understandings of the impact of the pandemic are refined. There is also an important opportunity to consider the limitations of the research available and identify what resources are needed to ensure future timely responses to major disruptions to our way of life to understand the mental health impacts.

## Supplementary Information

**Additional file 1:** **Table 1** Research conducted among general Australian adult population [37, 47]. **Table 2** Research conducted among specific subgroups in the population.

**Additional file 2. **PRISMA-ScR Checklist.

## Data Availability

Data sharing is not applicable to this article as no datasets were generated or analysed during the current study.

## Footnotes

1 https://www.srcentre.com.au/services/life-in-australia-panel

2 https://melbourneinstitute.unimelb.edu.au/data/COVID-19-tracker

3 https://www.monash.edu/medicine/living-with-COVID-19-restrictions-survey

4 https://www.researchgate.net/project/COVID-19-and-you-mentaL-heaLth-in-AusTralia-now-survEy-COLLATE-project

5 https://www.maprc.org.au/COVID19-mental-health
