# Supplementary material for: COVID-19 and mental health in Australia – a scoping review
Source: BMC Public Health. 2022 Jun 15;22:1200. doi: 10.1186/s12889-022-13527-9 (PMC9200373; doi:10.1186/s12889-022-13527-9)
Supplement: Supplementary file 1 — Additional file 1: Table 1 Research conducted among general Australian adult population [37, 47]. Table 2 Research conducted among specific subgroups in the population. [file 12889_2022_13527_MOESM1_ESM.docx]

## Table 1. Research conducted among general Australian adult population.

| **Authors (year)** | **Document type** | **Sample size and representative** | **Timing** | **Pre-Covid comparison?** | **Key mental health outcomes** | **Validated mental health measure?** | **Mental health compared to pre-COVID-19** | **Main risk or protective factors** |
| --- | --- | --- | --- | --- | --- | --- | --- | --- |
| Biddle, Edwards & Gray et al. (2020a) | Report | 3249.  Nationally representative (ANUpoll) | April 2020 and May 2020 | Yes (Feb 2017, ANUpoll) | Psychological distress | Yes (Kessler 6) | Significant increase between February 2017 and April 2020, and minor decrease between April and May 2020 | *Risks*: Aged ≤45, poorer quality relationships, poorer employment or financial status, stress, poorer future outlook, and COVID-related loneliness, anxiety or worry. |
| Biddle, Edwards & Gray et al. (2020b) | Report | 3155.  Nationally representative (ANUpoll) | April 2020 | Yes (Feb 2017, ANUpoll) | Psychological distress | Yes (Kessler 6) | Significant increase. | As above. |
|  |  |  |  | Yes (Feb 2020, ANUpoll) | Subjective wellbeing | No (Likert scale questions) | Significant decrease. | Age (younger adults less wellbeing) |
|  |  |  |  | Yes (Feb 2020, ANUpoll) | Social cohesion and trust | No (Likert question) | Significant increase. | Age (older adults greater social cohesion) |
| Botha, Butterworth & Wilkins (2020) | Report | 20906.  Nationally representative (TTPN-Taking the Pulse of the Nation Survey) | Every week from April 2020 to November 2020 | Yes (HILDA-Household, Income and Labour Dynamics in Australia Survey, 2017) | Mental distress | No (Likert question, based on Kessler 10) | Significant increase from pre-COVID.  Decreased April-May and then increased June-Oct | *Risk*: Number of COVID-cases (population-level), Financial stress. |
|  |  |  |  |  | Financial stress | No (Likert question) | Increased March-April, decreased April-Aug, increased Aug-Oct | Linked to government support. |
| Dawel, Shou & Smithson et al. (2020) | Journal article | 1296.  Nationally representative (The Australian National COVID-19 Mental Health, Behaviour & Risk Communication (COVID-MHBRC Survey) | March 2020 | Yes (not the same study, international samples) | Depression | Yes (Patient Health Questionnaire-9) | Elevated relative to norms. | *Risks*: Being a younger adult, female, having a current mental health disorder, and COVID-related work impairment, social impairment, or financial distress. Direct exposure to COVID-19 was not a significant risk factor. |
|  |  |  |  |  | Anxiety | Yes (Generalized Anxiety Disorder-7) | Elevated relative to norms. |  |
|  |  |  |  |  | General psychological wellbeing | Yes (World Health Organization Wellbeing Index-5) | No comparisons |  |
| Du, Yang, King et al. (2020) | Journal article | Not applicable (Internet search volumes in Google Trends).  Not representative | January 2020 to March 2020 | No | Fear-related emotions | No (number of searches for key words "fear", "panic", "worry) | Mediated the relationship between COVID-19 cases and other searches. |  |
|  |  |  |  |  | Protective behaviour | No (number of searches) | Sig, associated with COVID-19 cases. |  |
|  |  |  |  |  | Health-related knowledge | No (number of searches) | Sig, associated with COVID-19 cases. |  |
|  |  |  |  |  | Panic buying | No (number of searches) | Sig, associated with COVID-19 cases. |  |
| Ewing & Vu (2020) | Journal article | Not applicable (10421 tweets).  Not representative | April 2020 to May 2020 | No | Home schooling | No (tweets related to home schooling) | Over 3 weeks, tweets became sig. more negative, less humorous, more appreciative of teachers, less aimed at government, and more about the impacts of remote learning. |  |
| Fisher, Tran & Hammarberg et al. (2020) | Journal article | 13829.  Not representative | April 2020 to May 2020 | Yes (not the same study, other Australian samples) | Depression | Yes (Patient Health Questionnaire-9) | Elevated relative to norms. | *Risks*: Direct exposure to COVID-19, job loss, and finding COVID-19-related restrictions difficult. |
|  |  |  |  |  | Suicidality or self-harm |  | Elevated relative to norms. |  |
|  |  |  |  |  | Anxiety | Yes (Generalised Anxiety Disorder Scale-7) | Elevated relative to norms. |  |
|  |  |  |  |  | Irritability |  | Elevated relative to norms. |  |
|  |  |  |  |  | Optimism | No (Likert question) | Relatively optimistic |  |
| Gurvich, Thomas & Thomas et al. (2020) | Journal article | 1495.  Not representative | April 2020 to May 2020 | No | Psychological impact of COVID (PTSD symptoms) | Yes (Impact of Event Scale-Revised) | Unclear | *Risks*: Being female, self-blaming, venting, behavioural disengagement and self-distraction.  *Protective*: Positive reframing, acceptance, and humour. |
|  |  |  |  |  | Depression | Yes (Depression Anxiety Stress Scales) |  |  |
|  |  |  |  |  | Anxiety |  |  |  |
|  |  |  |  |  | Stress |  |  |  |
|  |  |  |  |  | Suicidal thoughts | Yes (Beck Depression Inventory) |  |  |
| Newby, O’Moore, Tang et al. (2020) | Journal article | 5070.  Not representative | March 2020 to April 2020 | Yes (Normative data from Australia and elsewhere) | Depression | Yes (Depression Anxiety Stress Scales) | High relative to norms. | *Risks*: Being younger, being less well educated, chronic illness, mental health diagnosis, self-rated poorer health, being a student or at home parent (rather than in paid employment), concern/worry about contracting COVID-19, concern/worry about loved ones contracting COVID-19, self-rated likelihood of contracting COVID-19, perceived control et al. |
|  |  |  |  |  | Anxiety |  | High relative to norms. |  |
|  |  |  |  |  | Stress |  | High relative to norms. |  |
|  |  |  |  | Yes (Self-report changes since outbreak) | General mental health | No (Likert question) | Worsened |  |
|  |  |  |  | No | Loneliness | No (Likert question) | Unclear |  |
|  |  |  |  | No | Worry about finances | No (Likert question) | Unclear |  |
|  |  |  |  | No | Anxiety about the future | No (Likert question) | Unclear |  |
|  |  |  |  | Yes (2007 Australian national data) | Health anxiety severity | Yes (Whiteley-6) | Elevated relative to general expectations. |  |
|  |  |  |  | No | Contamination fears, washing behaviours | Yes (Padua Inventory of Obsessions and compulsions) | Unclear |  |
| Owen, Tran & Hammarberg (2020) | Journal article | 13829.  Not representative | April 2020 to May 2020 | No | Bothersome poor appetite or overeating | Yes (Patient Health Questionnaire-9) | Prevalent | *Risks*: Anxiety, in lockdown with children, severely impacted by lockdown, concern about contracting COVID-19. |
| Rossell, Neill & Phillipou et al. (2020) | Journal article | 5158.  Not representative | April 2020 | Yes (Australian norms from 1995) | Negative emotions | Yes (Depression Anxiety Stress Scales-21) | Significantly elevated relative to norms. | *Risks*: Being female, in a state with fewer COVID-19 cases, mental health history, financial stress, affected by restrictions, expect longer duration of pandemic. |
| Stanton, To & Khalesi et al. (2020) | Journal article | 1491.  Not representative | April 2020 | No | Depression  Anxiety  Stress | Yes (Depression Anxiety Stress Scales-21) | Unclear | *Risks*: Being female, single, low physical activity, poor sleep, alcohol use, chronic disease. Additionally, for depression, being younger, low education, low income. |

Note: Results reported as ‘unclear’ included no robust information regarding pre-COVID comparisons and/or were focused on comparisons/risks within the sample.

## Table 2 Research conducted among specific subgroups in the population.

| **Authors (year)** | **Document type** | **Sample size and population of interest** | **Timing** | **Pre-Covid comparison?** | **Key mental health outcomes** | **Validated mental health measure?** | **Mental health outcome compared to pre-Covid** | **Main risk or protective factors** |
| --- | --- | --- | --- | --- | --- | --- | --- | --- |
| Broadway, Méndez & Moschion (2020) | Report | 6831 observations^1^ of parents aged 25-64 of children aged 0-18 years old (Taking the Pulse of the Nation survey) | June 2020 to August 2020 | Yes (HILDA - The Household, Income and Labour Dynamics in Australia for 2017) | Mental distress | No (Likert scale question, based on Kessler 10) | Significant increase. | *Risks*: Being a parent, fathers, employed while also being a parent, partner not being employed and financial distress, younger child age. |
| Cheek, Craig & West et al.(2020) | Journal article | Admissions data. Paediatric patients in four urban hospitals in Victoria | March 2020 to May 2020 | Yes (presentations in the same period in 2019 and Feb-March 2020) | Presentations with a mental health diagnosis | No (administrative data) | Significant increase compared to 2019;  No change compared to February 2020. |  |
| Chivers, Garad & Boyle et al. (2020) | Journal article | Not applicable.  Posts on forums for expecting mothers in Australia | January 2020 to May 2020 | No | Analysis of posts. | No (Qualitative data) | Negative content and negative words are predominant. |  |
| Digby, Winton-Brown & Finlayson et al. (2020)^2^ | Journal article | 274 staff at one hospital in Melbourne | April 2020 to May 2020 | No (but qualitative comparisons pre-post COVID) | Analysis of responses to questions about working, concerns, and self-care during COVID-19. | No (Qualitative data) | Heightened worry about patient care; changes to job conditions and the hospital environment; pandemic impact; uncertainty and isolation; management and leadership; more staff support needed |  |
| Dobson, Malpas & Burrell et al. (2021)^2^ | Journal article | 320 staff at one hospital in Melbourne | April 2020 to May 2020 | No | Depression | Yes (Patient health questionnaire-9) | High prevalence (21% moderate-to-severe depression) | *Risks*: Burnout, past psychiatric history, specific occupations.  *Protective*: Resilience. |
|  |  |  |  |  | Anxiety | Yes (Generalized anxiety disorder-7) | High prevalence (20% moderate-to-severe anxiety) |  |
|  |  |  |  |  | Likely PTSD | Yes (Impact of events scale-R) | High prevalence (29% moderate-to-severe PTSD) |  |
| Dragovic, Pascu & Hall et al.(2020) | Journal article | Not applicable.  Mental health presentations to emergency departments in WA. | January 2020 to May 2020 | Yes (presentations in the same period of 2019) | Anxiety and panic | No (number of presentations) | Significant increase. |  |
|  |  |  |  |  | Social and behavioural |  | Significant increase. |  |
|  |  |  |  |  | Suicidal and self-harm |  | Significant decrease. |  |
| Evans, Mikocka-Walus & Klas et al.(2020) | Journal article | 2130 parents (participants in the online-recruited Covid-19 Pandemic Adjustment Survey) | April 2020 | No | Qualitative thematic analysis of answers | No (Qualitative question) | Themes: 1) Boredom, depression, and suicide; 2) Families missing the things that keep them healthy; 3) Changing relationships: The push pull of intimacy; 4) Unprecedented demands of parenthood; 5) Unequal burden of Covid-19; 6) Holding on to positivity. |  |
| Griffiths, Sheehan & van Vreden et al. (2020) | Report | 2603 at 1^st^ wave; 1646 at 2^nd^; 1383 at 3^rd^; 1246 participated all three waves.^3^  Working Australian Adults (Covid-19 Work and Health Study) | Wave 1 (W1): March – June; Wave 2 (W2): April – July; Wave 3 (W3): July - Sept 2020 | No | Mental health | Yes (mental component summary scores of Short Form-12 quality of life questionnaire) | *Victoria*: improvement from W1 to W2, decline from W2 to W3;  *the Rest of Australia*: improvement W1 to W3.  *Vic vs. ROA at W3*: worse mental health in Vic (after accounting for W1). |  |
|  |  |  |  |  | Psychological distress | Yes (Kessler-6) | *Victoria*: reduction W1 to W2, increase W2 to W3;  *the Rest of Australia*: reduction from W1 to W3.  *Vic vs. ROA at W3*: Victorians more likely to have psychological distress (after accounting for W1). |  |
|  |  |  |  |  | Social isolation | Yes (The Duke Social Support Index) | *Victoria*: reduction of social isolation W1 to W2, sharp increase from W2 to W3;  *the Rest of Australia*: reduction from W1 to W3  *Vic vs. ROA at W3*: more social isolation in Vic (after accounting for W1). |  |
|  |  |  |  |  | Actions taken to manage mental health | No (multiple-choice questions) | More prevalent in Vic, compared to ROA during W3 (some items significant after accounting for W1) |  |
|  |  |  |  |  | Engagements with health services | No (multiple-choice questions) | More avoidance in Vic compared to ROA, during W3 (some items significant after accounting for W1) |  |
| Holton, Wynter & Trueman et al. (2020) | Journal article | 668 clinical hospital staff in Metropolitan Melbourne | May 2020 to June 2020 | Yes (Normative data from Australian population) | Psychological distress | Yes (Depression and anxiety stress scale -21) | Elevated relative to pre-COVID-19 population norms. | *Risks*: Direct contact with people with COVID-19, being a nurse or midwife.  *Protective*: Higher ratings of pandemic response and staff support strategies. |
| Ji, Basanovic & MacLeod et al. (2020) | Report | 132 Adults in strict self-isolation/ quarantine vs 2392 Adults undertaking social distancing (The COVID-19 Care Study) | April 2020 to June 2019 | No | Impact of COVID-19 on quality of life | No (multiple-choice and Likert scale questions) | Negative impacts higher in quarantine/self-isolation group (62% vs 56% rated negative) | *Risks for quarantine /self-isolation group*: Mandatory quarantine, living in quarantine facility.  *Risks for social distancing group*: Male, current mental health condition, not living with kids <18. |
|  |  |  |  |  | Concern about negative outcomes in general |  | Higher in quarantine/self-isolation group (44% vs 28% very or extremely concerned) | *Risks for social distancing group*: Female, current mental health conditions, young, student, unemployed |
|  |  |  |  |  | Worry about being isolated and lonely |  | Frequent (34% vs 20%) | *Risks for both groups*: current mental health conditions |
|  |  |  |  |  | Worry about experiencing anxiety/stress |  | Frequent (29% vs 24%) |  |
|  |  |  |  |  | Worry about becoming unmotivated or listless |  | Frequent (29% vs 22%) |  |
|  |  |  |  |  | Silver linings |  | Common (4 silver linings on average for both groups) | *Protective for social distancing group:* Female, higher educated, did not live alone |
| Johnston, Mohammed & Van Der Linden (2020) | Journal article | 3676 households with a child aged <15 (pre-stratified to approx. a nationally representative sample) | April 2020 to July 2020 | No | Self-rated mental health | No (Likert question) | No gender differences |  |
| Karantzas, Chesterman & Ferguson et al. (2020) | Pre-print | 1829 partnered parents (online-recruited Covid-19 Pandemic Adjustment Survey) | April 2020 | No | Relationship quality | Yes (Perceived relationship quality component) | COVID-19 stressors were sign. associated with poorer relationship quality. | *Protective*: Relationship adaptation |
|  |  |  |  |  | Loneliness | Yes (UCLA Loneliness Scale) | No significant association between COVID-19 stressors and loneliness | *Protective*: Relationship adaptation |
| Leske, Kõlves & Crompton et al. (2020) | Journal article | Register/admin data.  Individuals who had died by suicide in Queensland (interim Queensland Suicide Register) | Data spanning 2015 - 2020 | Yes (data divided by 29 Jan 2020) | Monthly suspected suicide rate | No (administrative data) | No change. |  |
|  |  |  |  |  | Motives related to recent unemployment, financial problems, domestic violence or relationship breakdown | No (report from police) |  |  |
| Li, Beames & Newby et al. (2020) | Pre-print | 760 Australian adolescents (aged 12-18 years recruited via social media) | June 2020 to August 2020 | Yes (Normative data from Australian and international samples prior to the pandemic) | Psychological distress | Yes (Kessler-6) | Elevated relative to norms. | *Risk*: History of depression and/or anxiety |
|  |  |  |  |  | Mental wellbeing | Yes (Warwick Edinburgh Mental Wellbeing Scale) | Elevated relative to norms. |  |
|  |  |  |  |  | Health anxiety | Yes (Body Preoccupation Scale of the Illness Attitude Scales) | Elevated relative to norms. |  |
| Lyons, Wilcox & Leung et al. (2020) | Journal article | 297 medical students from University of Western Australia |  | No | Psychological distress | Yes (Kessler-10) | Moderate level |  |
|  |  |  |  | Yes (Self-report changes since the outbreak) | Change in mental well-being since Covid-19 onset | No (Likert question) | Widespread (reported by 68%) |  |
| Mikocka-Walus, Stokes & Evans et al. (2020) | Pre-print | 2110 parents of children aged 0-18 (Covid-19 Pandemic Adjustment Survey) | April 2020 | No | Stress | Yes (Depression and anxiety stress scale -21) | Unclear | *Risk:* Loneliness.  *Protective:* Resilience, partner support |
|  |  |  |  |  | Anxiety |  |  |  |
|  |  |  |  |  | Depression |  |  |  |
| Munasingh, Sperandei & Freebairn et al. (2020) | Journal article | 582 adolescents (aged 13-19 recruited via social media from the general Sydney population) | November 2019 to April 2020 | Yes (before and after 23 March) | Psychological distress | Yes (Kessler-6) | Moderate increase. |  |
|  |  |  |  |  | Positive psychological characteristics | Yes (Engagement, Perseverance, Optimism, Connectedness, and Happiness measure) | Significant decrease. |  |
|  |  |  |  |  | Social relationships (alone in past hour) | No (multiple-choice questions) | Significant increase. |  |
| Oliva & Johnston (2020) | Journal article | 384 adults living alone with or without a pet | May 2020 | No | Loneliness | Yes (UCLA Loneliness Scale) | Unclear | *Protective:* Dog ownership, mindfulness |
|  |  |  |  |  | Experience of COVID-19 and pet ownership | No (Multiple choice. qualitative questions) |  |  |
| Olive, Sciberras, Berkowitz et al. (2020) | Pre-print | 2365 parents of children aged 0-18 years (Covid-19 Pandemic Adjustment Survey) | April 2020 | No | Parent mental health | Yes (Depression and anxiety stress scale -21) | Unclear | *Risks for children:* Weekend screen time, sleep disturbance, physical activity disturbance.  *Risks for parents:* Less physical activity and reduced sleep quality. |
|  |  |  |  |  | Parent emotion regulation | Yes (Difficulties in Emotion Regulation Scale) |  |  |
|  |  |  |  |  | Parent affect | Yes (Positive and Negative Affect Schedule Short Form) |  |  |
|  |  |  |  |  | Child depression^4^ | Yes (Short Mood and Feelings Questionnaire) |  |  |
|  |  |  |  |  | Child anxiety^4^ | Yes (Modified Brief Spence Children’s Anxiety Scale) |  |  |
|  |  |  |  |  | Irritability | No (Likert questions) |  |  |
| Phillipou, Meyer & Neill et al. (2020) | Journal article | 180 individuals with eating disorder vs 5289 from general population (COvid-19 and you: mentaL heaLth in AusTralia now survEy: COLLATE) | April 2020 | No | Experience of mental illness | No (Likert questions) | Unclear | *Risk:* Having an eating disorder |
|  |  |  |  | No | Current negative mood states | Yes (Depression and anxiety stress scale -21) |  |  |
|  |  |  |  | Yes (Self-report) | Eating and exercise behaviours | Yes (Eating Disorders Examination Questionnaire) |  |  |
| Pikoos, Buzwell & Sharp et al. (2020) | Journal article | 216 individuals from general public with different levels of dysmorphic concern (recruited from social media) | May 2020 | Yes (Self-report comparisons between prior to the Covid-19 pandemic and at the peak of Covid-19 restrictions) | Dysmorphic concern | Yes (the Dysmorphic Concern Questionnaire) | Unclear | *Risks*: Living alone, younger, higher dysmorphic concern. |
|  |  |  |  |  | Appearance-focused behaviours | No (adaptation of features from the DSM-5) |  |  |
|  |  |  |  |  | Negative emotional states | Yes (Depression and anxiety stress scale -21) |  |  |
|  |  |  |  |  | Distress at beauty service disruption | No (Likert questions) |  |  |
| Rahman, Hoque & Alif et al. (2020) | Journal article | 587 Australian adults recruited from GP and Allied Healthcare settings, community groups, or online | June 2020 | No | Psychological impact | Yes (Kessler-10) | Unclear | *Risks:* Prior mental health condition, increased smoking and alcohol drinking, high fear levels, being female, changed work status, providing care to known or suspected COVID-19 cases. |
|  |  |  |  |  | Fear of COVID-19 | Yes (Fear of COVID-19 Scale) |  |  |
|  |  |  |  |  | Coping strategies | Yes (Brief Resilient Coping Scale) |  |  |
| Shaban, Nahidi & Sotomayor-Castillo et al. (2020) | Journal article | 11 First patients with confirmed COVID-19 in a designated isolation facility in NSW |  | No | "Knowing about COVID-19"; "Planning for a response to COVID-19"; "Being infected"; "Life in isolation, and the room"; "Post-discharge life". | No (Qualitative interviews) | Both positive and negative experiences. | *Protective*: Context of social environment and individual's resources |
| Sollis, Biddle & Edwards et al. (2020) | Report | 3155 Australians who participated in COVID-19-related surveys (ANUpoll) | April 2020 | No | Distress and satisfaction from research participation | No (Likert questions) | No impact of research participation on distress and/or satisfaction. |  |
|  |  |  |  |  | Subjective wellbeing relating to completing the survey. | No (Likert questions) | Negative impact for a sub-population. | *Risk*: Mental health concerns or living in financial insecurity |
| Staples, Nielssen & Kayrouz et al. (2020)^5^ | Journal article | 5454 people accessing digital mental health service (MindSpot Clinic) | March 2020 to June 2020 | Yes (data collected at September 2019) | Psychological distress | Yes (Kessler-10) | No change. |  |
|  |  |  |  |  | Anxiety | Yes (Generalized Anxiety Disorder-7) | Some acute elevation, but returned to baseline. |  |
|  |  |  |  |  | Depression | Yes (Patient Health Questionnaire-9) | No change. |  |
|  |  |  |  |  | Emerging difficulties with anxiety and depression. | No (Likert questions) | Increase. |  |
|  |  |  |  |  | Suicidal ideation and intent. | No (Likert questions) | No change. |  |
| Titov, Staples & Kayrouz et al.(2020) | Journal article | 1668 people accessing digital mental health service (MindSpot Clinic) | March 2020 to April 2020 | Yes (data collected at September 2019) | Psychological symptoms | Yes (Kessler-10) | No change. | *Risks:* Being female, or being unemployed. |
|  |  |  |  |  | Anxiety | Yes (Generalized Anxiety Disorder-7) | Small but significant increase |  |
|  |  |  |  |  | Depression | Yes (Patient Health Questionnaire-9) | No change. |  |
|  |  |  |  |  | Website visits and number of calls made to the MindSpot Clinic. | No (Likert questions) | Significant large increase. |  |
|  |  |  |  |  | Emerging difficulties with anxiety and depression. | No (Likert questions) | Significant increase. |  |
|  |  |  |  |  | Suicidal ideation and intent. | No (Likert questions) | No change. |  |
| Van Agteren, Bartholomaeus & Fassnacht et al. (2020) | Journal article | 673 (COVID-19 group) vs 1264 (pre-COVID general group) vs 340 (pre-COVID help seeking group).  Adults engaged with services offered by the South Australian Health and Medical Research Institute Wellbeing and Resilience Centre | Since March 29 2020 | Yes (data collected between February 2019 - February 2020) | Psychological distress. | Yes (Depression and anxiety stress scale -21) | Significantly worse than both pre-COVID groups |  |
|  |  |  |  |  | Well-being. | Yes (Mental Health Continuum Short-Form, and Satisfaction With Life Scale) | Significantly worse than both pre-COVID groups |  |
|  |  |  |  |  | Adaptive states. | Yes (Brief Resilience Scale) | Significantly worse than both pre-COVID groups |  |
| Van Rheenen, Meyer & Neill et al. (2020) | Journal article | 1292 with a mood disorder vs 3167 without any mental disorder, identified in COvid-19 and you: mentaL heaLth in AusTralia now survEy (COLLATE) | April 2020 | No | Psychological distress. | Yes (Depression and anxiety stress scale -21) | Elevated in those with mood disorders | *Risks:* Maladaptive situational and lifestyle changes in response to COVID-19 |
|  |  |  |  |  | Mental health history. | No (multiple choice and Likert questions) |  |  |
| Westrupp, Stokes & Fuller-Tyszkiewicz et al. (2020) | Pre-print | 2365 Australian parents of children aged 0-18 years (Covid-19 Pandemic Adjustment Survey) | April 2020 | Yes (a subsample of parents 17529) from the Australian Unity Wellbeing Index, collected 2002-2019) | Subjective wellbeing. | Yes (Personal wellbeing index) | Significant decrease. | *Risks:* Socially disadvantaged, pre-existing mental health difficulties, and COVID-19 related work changes. |
| Westrupp, Bennett, Berkowitz et al. (2020) | Pre-print | 2365 Australian parents of children aged 0-18 years (Covid-19 Pandemic Adjustment Survey) | April 2020 | Yes (pre-COVID studies, Child and Parent Emotional Study, Longitudinal Study of Australian Children) | Parent depression, anxiety, stress. | Yes (DASS-21) | Significant increase | *Risks:* Pre-existing mental health conditions, pre-existing financial deprivation. |
|  |  |  |  |  | Parent emotion regulation. | Yes (Difficulties in Emotion Regulation Scale) | Unclear |  |
|  |  |  |  |  | Child depression.^4^ | Short Mood and Feelings Questionnaire | Unclear |  |
|  |  |  |  |  | Child anxiety.^4^ | Yes (Modified Brief Spence Children’s Anxiety Scale) | High |  |
|  |  |  |  |  | Parenting Irritability. | No (Likert questions) | Increase |  |
|  |  |  |  |  | Couple verbal conflict. | No (Likert question) | Unclear |  |
|  |  |  |  |  | Family expressiveness. | No (Likert questions) | Lower |  |

^1^ Of them, 3422 observations had data on partner’s employment status.

^2^ Digby et al.(2020) and Dobson et al. (2020) reported the results from the same study. Digby et al. (2020) reported the participants’ responses on qualitative questions, while Dobson et al. (2020) reported the quantitative data.

^3^ Of the 1246 participants, one third were from Victoria, the others were from the rest of Australia.

^4^ Child depression and anxiety were measured among Children ≥2 years of age.

^5^ Staples et al. (2020) is a follow-up study on Titov et al. (2020).

Note: Results reported as ‘unclear’ included no robust information regarding pre-COVID comparisons/norms and were often focused on comparisons/risks within the sample.
